# Anaerobic gut bacteria and their potential role in the initiation, exacerbation, and development of human colorectal cancer: a narrative review

**DOI:** 10.3389/fcimb.2025.1559001

**Published:** 2025-07-25

**Authors:** Sahar Sabour, Hanieh Sadeghi Koupaei, Hadi Ghasemi, Mansour Amin, Taher Azimi

**Affiliations:** ^1^ Infectious and Tropical Diseases Research Center, Health Research Institute, Ahvaz Jundishapur University of Medical Sciences, Ahvaz, Iran; ^2^ Department of Microbiology, Faculty of Medicine, Ahvaz Jundishapur University of Medical Sciences, Ahvaz, Iran; ^3^ Pediatric Congenital Hematologic Disorders Research Center, Mofid Children Hospital, Shahid Beheshti University of Medical Sciences, Tehran, Iran; ^4^ HIV/AIDS Research Center, Institute of Health, Shiraz University of Medical Sciences, Shiraz, Iran; ^5^ Department of Bacteriology & Virology, School of Medicine, Shiraz University of Medical Sciences, Shiraz, Iran

**Keywords:** colorectal cancer, CRC, anaerobic gut bacterial, gut bacterial, narrative review

## Abstract

Colorectal cancer (CRC) is known as the second leading cause of cancer-related deaths around the world. Rectal bleeding, changes in bowel movements, weight loss, and fatigue are the main clinical presentations of CRC. While the exact etiology of the disease is unknown, CRC is considered a complex and multifactorial disease resulted from an intricate interplay of genetic and environmental factors. Moreover, CRC is known as a chronic inflammation–associated tumor, and patients with inflammatory bowel disease (IBD) or Irritable bowel syndrome (IBS) are susceptible groups to CRC development. The gut microbiota and its metabolites play a crucial role in the development and progression of CRC. CRC can be created after anaerobic bacterial infections such as Enterotoxigenic *Bacteroides fragilis* (ETBF), *Fusobacterium*, *Clostridium difficile*, *Clostridium perfringens*, *Clostridium septicum*, *Peptostreptococcus*, *Prevotella*, *Veillonella*, etc. Activation of Wnt signaling, loss of tissue architecture, proinflammatory signaling, and genotoxic cellular DNA damage are the primary mechanisms by which anaerobic bacteria induce carcinogenesis in CRC. Besides, spore germination and toxin production are done in hypoxic and acidic conditions. Therefore, according to the presence of this condition (anaerobic glycolysis) in tumor tissue, the tumor environment is suitable for the formation of anaerobic infections. The current review‐based study aims to discuss the important aspects of these mechanisms and their possible roles in the initiation, development, and exacerbation of CRC.

## Introduction

Colorectal cancer (CRC) is the third most common cancer around the world (Bray et al., 2024; Cao et al., 2024; Hong et al., 2024). Despite the existence of different treatment regimens, CRC ranks as the second leading cause of cancer-related deaths with an estimated 3.2 million new cases and 1.6 million deaths in 2024 (BGI Genomics, 2024). Rectal bleeding, changes in bowel movements, weight loss, and fatigue are the primary clinical presentations of CRC (Koliarakis et al., 2024). CRC is a multifactorial disease, and its etiology is not fully determined. It is presumed that genetic and environmental factors are linked to the initiation, progression, and exacerbation of CRC (Cheng et al., 2024). Indeed, CRC is known as a chronic inflammation–associated tumor, and patients with ulcerative colitis or Crohn’s disease (inflammatory bowel disease) are susceptible groups to CRC development (Eskandari-Malayeri et al., 2024).

Globally, previously published studies have stated that CRC can be triggered by anaerobic bacterial infections, including Enterotoxigenic *Bacteroides fragilis* (ETBF), *Fusobacterium nucleatum*, *Clostridium difficile*, *C. perfringens*, *C. septicum*, *Peptostreptococcus*, *Prevotella*, and *Veillonella* (Kajihara et al., 2023; Conde-Pérez et al., 2024; Hong et al., 2024). The gut microbiota and its metabolites play a crucial role in the development and progression of CRC (Cheng et al., 2024). Disruptions in microbiota composition are termed dysbiosis (Shariati et al., 2019a; Azimi et al., 2020). Microbiota-related carcinogenesis is primarily caused by dysbiosis-related inflammation and carcinogen formation (Azimi et al., 2018; Shariati et al., 2019b). Moreover, secondary bile acids (BAs) and short-chain fatty acids (SCFAs), which are two main bacterial metabolites in the colon, play either protective or harmful roles in the initiation and development of CRC (Cheng et al., 2024). Activation of Wnt signaling, loss of tissue architecture, proinflammatory signaling, and genotoxic cellular DNA damage are the primary mechanisms by which anaerobic bacteria induce carcinogenesis in CRC (Hong et al., 2024). The role and possible mechanisms of anaerobic pathogenic bacteria are summarized in **Table 1**. According to what was mentioned earlier and with respect to possible roles of anaerobic bacteria in cancer development and progression, the current review‐based study aims to discuss their potential roles and important aspects of mechanisms in the initiation, development, and exacerbation of CRC.

**Table 1:** The possible mechanisms of anaerobic gut bacteria implicated in the initiation, exacerbation, and development of human CRC.

Gut pathogens		Possible mechanisms	Gut pathogens		Possible mechanisms
1	*Fusobacterium nucleatum*	• Interaction of the FadA with E-cadherin upregulates the Annexin A1 expression • Activation of the Wnt/β-catenin signalling pathway • Breaks of DNA Double-Strand • Fap2 interaction with D-galactose-β (1-3)-N-acetyl-D-galactosamine • Interaction of LPS with TLR-4 • Fap2 binds to the human immune inhibitory receptor T-cell immunoglobulin and ITIM domain • Inhibits NK cytotoxicity and modulating the tumor-immune microenvironment • Formation of bacterial co-aggregation and biofilm • Promotes the M2 polarization of macrophages and leads to immunosuppression in the tumor microenvironment *• F. nucleatum* can consume butyric acid, significantly decreasing the concentration of butyrate in CRC cells and promoting CRC development *• F. nucleatum* upregulates the expression levels of BIRC3 in CRC cells and promotes chemoresistance to chemotherapy • Ppplies the miR18α, miR4802, MYD88 TLR-4, and unc-51-like kinase 1/autophagy related 7 autophagy networks to create and control chemoresistance • Increase the expression of miR-21 • Activates the autophagy signals • inhibits the N6-adenosine-methyltransferase 70 kDa subunit enzyme • Induces the long non-coding RNA EVADR • Initiates the nuclear factor kappa-light-chain-enhancer of activated B cells • Binding of ADP-H to ALPK1 strongly activates NF-κB in intestinal epithelial cells and induces the expression of the inflammatory cytokine and anti-apoptotic genes • H2S produced by *F. nucleatum* disrupts the colonic epithelial cell barrier and induces free radical-related DNA damage • Overproduction of H2S promotes the proliferation of cancer cells in CRC • Activates the MAPK (JNK) - AP1 axis and up-regulates the MMP7 expression. • The overexpression of MMP7 on the cancer cell promotes metastasis in colon epithelial cells	6	*Clostridium perfringens*	• Long-term PPI use initiates and exacerbates the *C. perfringens* infection in the intestinal tract *• C. perfringens* enterotoxin could impair impairs Claudin-4, which forms tight junctions in colorectal cells and enhances cancer malignancy • Bacterium activates the YAP and promotes cancer progression
2	Enterotoxigenic *Bacteroides fragilis*	• BFT can induce the cleavage of the extracellular domain of the E-cadherin junction protein and lead to the disruption of E-cadherin and β-catenin linkage • Changes in the epithelial cell cytoskeletal structure and increases intestinal secretion and cell signalling via the β-catenin/Wnt pathway • Activates (NF-κB and promotes the production of inflammatory cytokines such as CXCL1 • Stimulate the secretion of IL-8 and c-myc expression • Activation of signal transducer and activator of transcription 3 • Produce the ROS and causes oxidative DNA damage • stimulates the high expression of IL-17 • ETBF down-regulates the miR-149-3p expression • ETBF can accelerate colitis and carcinogenesis related to DSS and azoxymethane.	7	*Clostridium septicum*	*• C. septicum* via DNA damage and defects in cellular DNA repair processes can lead to carcinogenesis in the colon • Hypoxia and acidity conditions in the tumor environment are suitable for spore germination, active *C. septicum* infection, and toxin production • Hyaluronidase, fibrinolysin, deoxyribonuclease, and hemolysins enable bacteria to metastasize and invade tissues in the colon
3	*Veillonella parvula*	*• V. parvula* used nitrate respiration to colonize the intestine during the inflammatory response • Increases B lymphocyte stimulator (BLyS) expression level and B-cell infiltration *• V. parvula* can exert an immunomodulatory effect in CRC cells	8	*Actinomyces israelii*	• This bacterium can disrupt the intestinal wall and enter the abdominal cavity • During the surgery for local recurrence, it's possible that *A. israelii* entered the abdominal cavity • Injuries to the oral cavity or gastrointestinal tract could have allowed organisms to enter the bloodstream
4	*Prevotella intermedia*	*• P. intermedia* metabolize the glucose for formate formation *• P. intermedia* induces colon cancer cell invasion and leads to metastatic dissemination in CRC *• P. intermedia* secretes the AMF molecule and ultimately promotes the CRC cell motility and metastasis	9	*Actinomyces odontolyticus*	• This bacterium secretes MVs and induces mitochondrial dysfunction and excessive ROS production • MVs specifically bind the TLR2 receptor and activate the NF-kB signaling pathway
5	*Clostridioides difficile*	• TcdB is an important toxin and has more toxic effects on colon cells • TcdB induces the inflammatory response, deregulates Rho-GTPases, and increases the expression levels of proto-oncogenes in colon • Expression of *PLC-γl is upregulated in CRC cells and* breast carcinoma • TcdA can induce apoptosis in PLC-γl-transformed cells • TcdA can upregulate the expression levels of BIM and activation of caspase-3 in PLC-γl-transformed cells	10	*Peptostreptococcus anaerobius*	• The interaction of *P. anaerobius* with TLR-2 and TLR-4 elevates ROS production in colon cells and leads to CRC tumorigenesis *• P. anaerobius* facilitates the activity of *SREBP-2* *• P. anaerobius* promotes cholesterol biosynthesis and enhances cell proliferation and tumorigenesis in CRC *• P. anaerobius* colonization increases MDSCs abundances in the tumor microenvironment • Recruitment of MDSCs into tumor microenvironment increases the chemoresistance of colorectal cancer • The accumulation of MDSCs in the tumour microenvironment can antagonistically affect antitumor CD8+ and CD4+ T cells *• P. anaerobius* stimulates the secretion of chemokine (C-X-C motif) ligand 1 • Interaction of PCWBR2 with epithelial cell receptor integrin α2/β1 leads to activation of NF-κB and ultimately promotes cell proliferation and pro-inflammatory immune responses in CRC cells
11	*Peptostreptococcus stomatis*	*• P. stomatis* can activate the ERBB2-MAPK) and promotes colonic tumorigenesis • Bacterium used its surface protein FBA to attach to the CRC cells • Attachment of *P. stomatis* to CRC cells leads to the activation of ERBB2 and downstream Ras/Raf/MAPK (MEK)-ERK cascades *• P. stomatis* can inactivate and inhibit the BRAF inhibitor and RTK inhibitor leading to the non-responsiveness to these drugs in CRC • Bacterium can suppress apoptosis and impair the gut barrier function		*-*	–

FadA, *Fusobacterium* adhesin A; Fap2, Fibroblast activation protein 2; LPS, Lipopolysaccharide; TLR-4, Toll-like receptor 4; NK cell, natural killer cell; BIRC3, Baculoviral IAP repeat-containing protein 3; EVADR, endogenous retroviral-associated adenocarcinoma lncRNA; ROS, reactive oxygen species; DSS, dextran sulfate sodium; YAP, Yes-associated protein; PPI, Proton pump inhibitors; MV, membrane vesicle; SREBP-2, *sterol regulatory element-binding protein 2*; MDSC, Myeloid-derived suppressor cell; ERBB-2, Erb-b2 receptor tyrosine kinase 2; MAPK, Mitogen-activated protein kinase; FBA, fructose-1,6-bisphosphate aldolase; ERK, extracellular signal-regulated kinase; RTK, receptor tyrosine kinase.

## Fusobacterium nucleatum

*Fusobacterium nucleatum* (*F. nucleatum*) is a spindle-shaped, non-spore-forming Gram-negative anaerobe bacterium. It is identified as a member of the human normal microbiota in oral cavity[12]. However, prior studies have indicated that *F. nucleatum* is an invasive, pro-inflammatory pathogen associated with various human disease conditions (Koliarakis et al., 2024).

**Figure 1:**
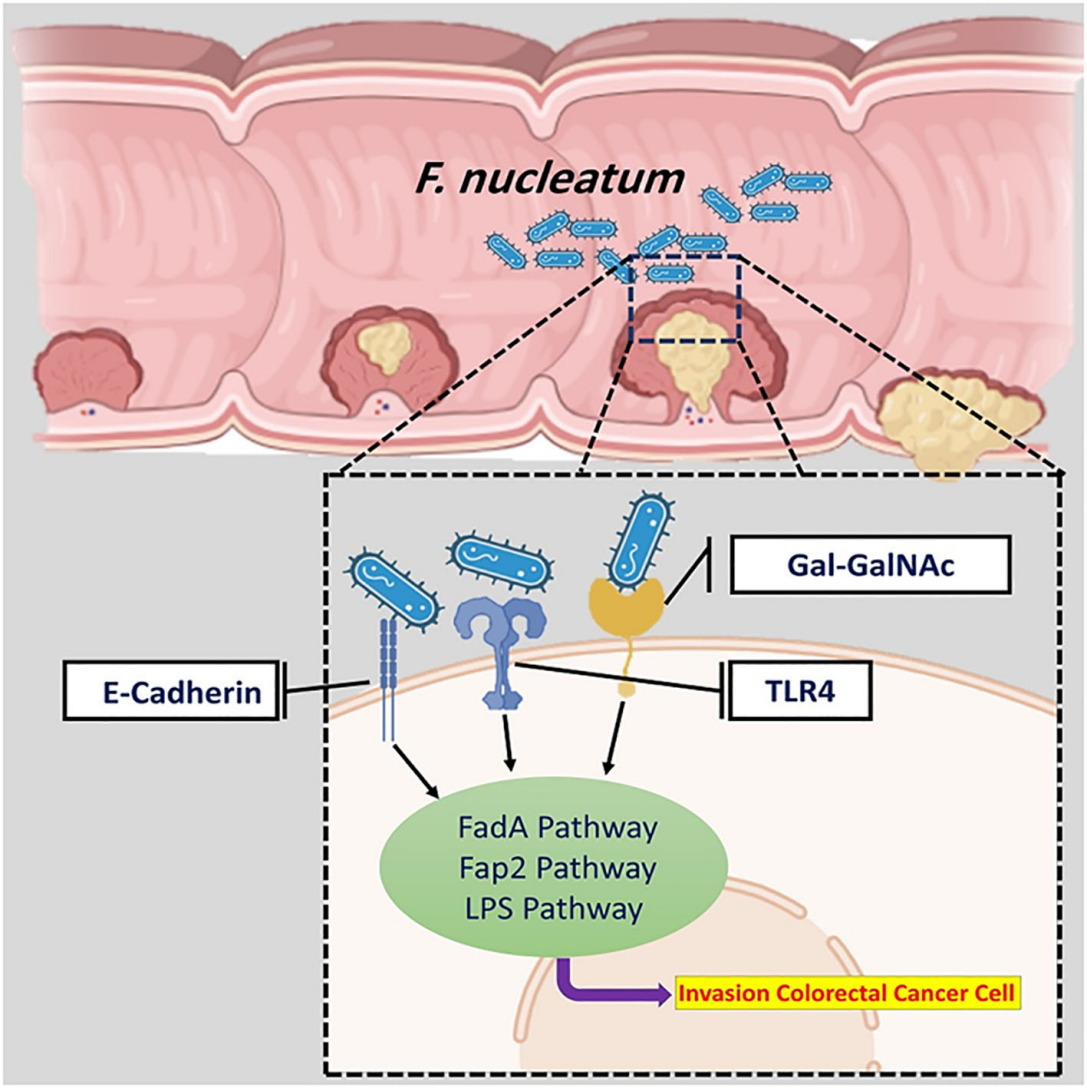
The overall mechanism of *F. nucleatum* in CRC. The entrance of *F. nucleatum* into colorectal tissues has been facilitated in three main ways.

*F. nucleatum* plays a key role in initiating and developing various cancers, such as liver, lung, and CRC (Liu et al., 2021). It has been discovered that different mechanisms of CRC are mediated by *F. nucleatum.* In most cases, the entrance of *F. nucleatum* into colorectal tissues has been facilitated in three main ways (**Figure 1**): 1) interaction of the effector *Fusobacterium* adhesin A (FadA) with E-cadherin, 2) fibroblast activation protein 2 (Fap2) interaction with D-galactose-β (1-3)-N-acetyl-D-galactosamine (Gal-GalNAc), and 3) interaction of Lipopolysaccharide (LPS) of *F. nucleatum* with Toll-like receptor 4 (TLR4) (Wang et al., 2023a; Martin-Gallausiaux et al., 2024).

**Figure 2:**
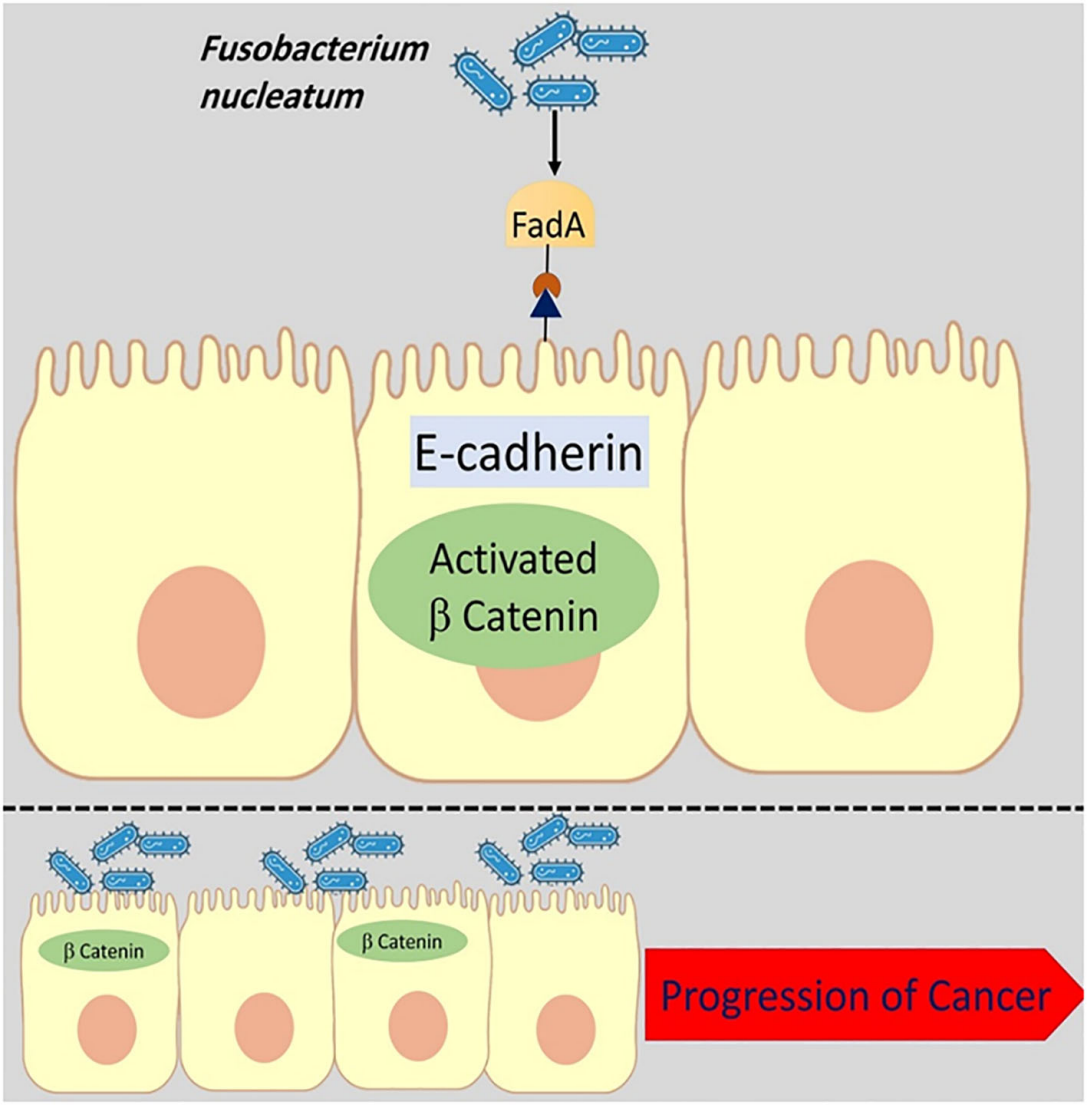
The binding of the FadA protein to E-cadherin upregulates the Annexin A1 expression and can stimulate carcinogenesis. The disruption of E-cadherin and β-catenin linkage leads to changes in the epithelial cell cytoskeletal structure and increases cancer progression.

Previously published studies have revealed overexpressed FadA gene levels in CRC patients’ faecal samples (Martin-Gallausiaux et al., 2024; Wang et al., 2024). Annexin A1 is a 35–40 kDa molecule and is considered as a main part of FadA protein. It is proposed that Annexin A1 can enable the FadA protein to influence different biological processes such as apoptosis, cell differentiation, cell proliferation, cell migration, and tumor growth (**Figure 2**) (Liu et al., 2024). The binding of the FadA protein to E-cadherin upregulates the Annexin A1 expression and can stimulate carcinogenesis (Cheng et al., 2024). Moreover, by activating the Wnt/β-catenin signalling pathway cyclin D1 (CycD1), it can induce and trigger oncogenic and inflammatory responses (Pignatelli et al., 2023). Interaction of Annexin A1 with epidermal growth factor receptor (EGFR) induces immune suppression in the tumor microenvironment (Araújo et al., 2021).

In addition, the interaction of *F. nucleatum* FadA protein with E-cadherin leads to DNSA damage (DNA Double-Strand breaks (DSBs)) and has a key role in cancer development (Guo et al., 2020).

**Figure 3:**
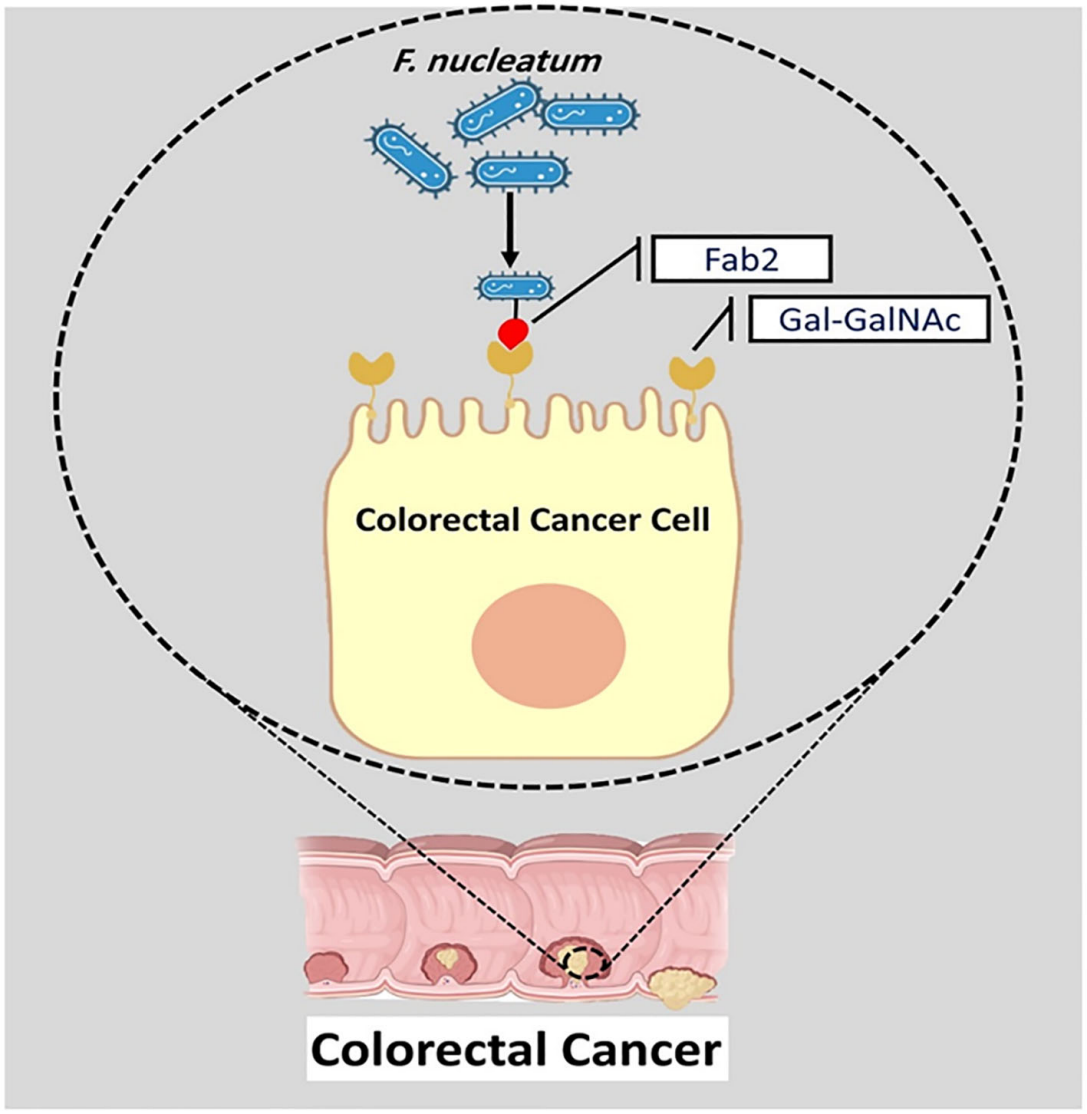
The interaction of Fap2 with tumor-specific sugar residue Gal-GalNAc promotes the attachment of *F. nucleatum* to biofilm and facilitates its localization and enrichment in CRC.

*F. nucleatum* Fap2 protein plays a significant role in attachment and instability of intestinal barriers. Fap2 can promote the entrance of *F. nucleatum* to intestinal epithelial cells (Luo et al., 2024). Furthermore, by interacting with tumor-specific sugar residue Gal-GalNAc, FadA and Fap2 proteins promote the attachment of *F. nucleatum* to biofilm and facilitate its localization and enrichment in CRC (**Figure 3**) (Zeddou, 2023). Fap2 binds to human immune inhibitory receptor T-cell immunoglobulin and ITIM domain, inhibiting natural killer cell (NK) cytotoxicity and modulating the tumor-immune microenvironment. Therefore, Fap2 can inhibit antitumor immunity in tumor microenvironment (Gur et al., 2015).

*F. nucleatum* has numerous adhesins on its surface, including RadD, Aid1, and FomA, that help in formation of bacterial co-aggregation and biofilm. In CRC, *F. nucleatum* dominant biofilms are a common finding, particularly when the cancer is on the right side of the colon (Liu et al., 2024).

M2 macrophages have a crucial role in tissue healing and inflammation. A previously published study stated that the interaction of *F. nucleatum* with a TLR4-dependent mechanism promotes the M2 polarization of macrophages. The promotion of M2 polarization of macrophages by *F. nucleatum* leads to immunosuppression in the tumor microenvironment (Chen et al., 2018; Mohammadi et al., 2022).

Butyrate is the main energy source for colonic cells and has anti-inflammatory and immunomodulatory effects. It inhibits tumor cell proliferation and migration, downregulates the Wnt signalling pathway, and limits tumor angiogenesis (Martin-Gallausiaux et al., 2024). Therefore, butyrate can inhibit tumor development in colonic cells. Wu et al. suggested that *F. nucleatum* can actively consume butyric acid, significantly reduce the concentration of butyrate in CRC cells, and promote CRC development. They presumed that *F. nucleatum* promotes the occurrence and development of CRC by reducing the population of butyrate-producing bacteria (Wu et al., 2024).

The combination of 5-Fluorouracil (5-Fu), irinotecan, or oxaliplatin is used as a standard treatment of advanced CRC (André et al., 2009). One of the primary causes of poor prognosis in CRC is chemoresistance. During the period of drug treatment, *F. nucleatum* upregulates the expression levels of Baculoviral IAP repeat-containing protein 3 (BIRC3) in CRC cells. An increase in expression levels of BIRC3 promotes chemoresistance to chemotherapy (Martin-Gallausiaux et al., 2024).

**Figure 4:**
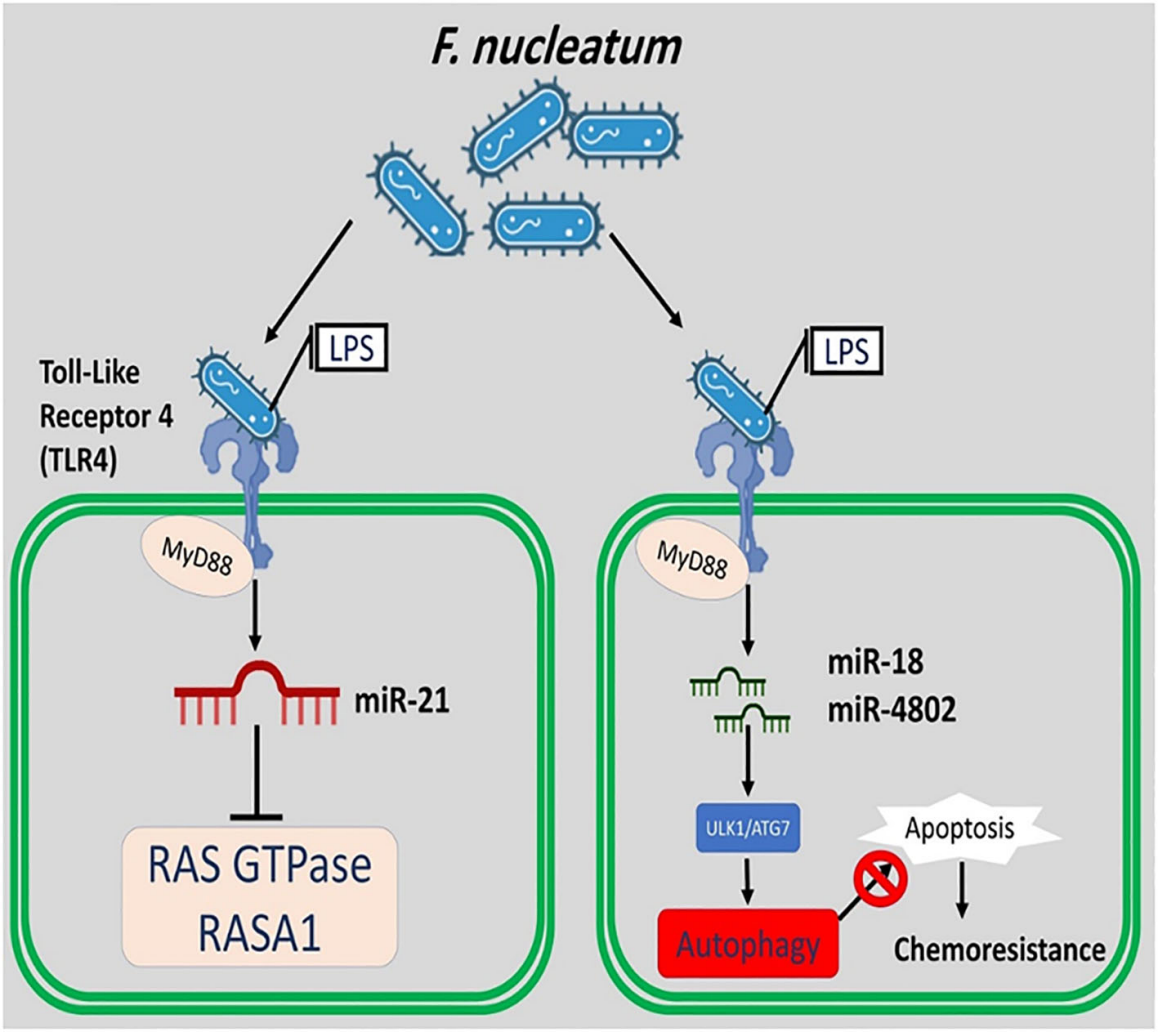
*F. nucleatum* applies the miR18, miR4802, MYD88, TLR-4, and unc-51-like kinase 1/autophagy related 7 autophagy networks to create and control chemoresistance in CRC cells. *F. nucleatum* induces the expression of miR-21 and suppresses the RAS GTPase and RASA1.

Moreover, *F. nucleatum* applies the miR18α, miR4802, MYD88 TLR-4, and unc-51-like kinase 1/autophagy related 7 autophagy networks to create and control chemoresistance in CRC cells (**Figure 4**) (Bostanghadiri et al., 2023).

It is revealed that *F. nucleatum* infection can promote CRC cell metastasis by several mechanisms. This bacterium activates the autophagy signals, inhibits the N6-adenosine-methyltransferase 70 kDa subunit (METTL3) enzyme, induces the long non-coding RNA endogenous retroviral-associated adenocarcinoma lncRNA (EVADR), and initiates the nuclear factor kappa-light-chain-enhancer of activated B cells (NF-κB) (Du et al., 2019; Chen et al., 2022; Liu et al., 2024).

ADP-heptose (ADP-H) is known as the pathogen-associated molecular pattern (PAMP) produced by *F. nucleatum* (Chen et al., 2022). Alpha kinase 1 (ALPK1) is the new pattern recognition receptor (PRR) that senses ADP-H. The binding of ADP-H to ALPK1 strongly activates nuclear factor-κB (NF-κB) in intestinal epithelial cells and induces the expression of inflammatory cytokines and anti-apoptotic genes (Milivojevic et al., 2017; Martin-Gallausiaux et al., 2022). It is revealed that the binding of ADP-H to ALPK1 increases the expression levels of IL-6, IL-8, IL-17, TNF-α, TLR-2, and TLR-4 genes in CRC cells. Moreover, this process reduces the chemosensitivity of CRC cells (Martin-Gallausiaux et al., 2024).

Previously published studies have identified a significant relationship between miR-21 expression and high levels of *F. nucleatum* in CRC cells. The expression of miR-21 is upregulated by hyperactive NF-kB binding to its promoter in CRC cells. This finding is partial support for the role of *F. nucleatum* in carcinogenesis by inducing miR-21. It is possible that *F. nucleatum* induces the expression of miR-21 and suppresses the Ras p21 protein activator 1 (RASA1) (a regulator of Ras GDP and GTP) (Bostanghadiri et al., 2023).

Both hydrogen sulfide (H2S) and sulfur-containing amino acids can initiate or promote the development of various cancers, such as CRC (Kim et al., 2016). *F. nucleatum* metabolizes sulfur-containing amino acids and produces H2S. Moreover, this bacterium can produce H_2_S from L-cysteine. H2S produced by *F. nucleatum* disrupts the colonic epithelial cell barrier and induces free radical-related DNA damage. It is revealed that the overproduction of H2S promotes the proliferation of cancer cells in CRC patients (Wang et al., 2023b).

Matrix metalloproteinase 7 (MMP7) is a significant member of the MMPs family and has a significant role in metastasis, tumor growth, and angiogenesis (Pezeshkian et al., 2021). MMP7 played a vital role in the activation of other MMPs, such as pro-MMP2 and pro-MMP9. It is revealed that *F. nucleatum* activates the MAPK (JNK) - AP1 axis (a transcription factor which targets MMP7) and upregulates the MMP7 expression. The overexpression of MMP7 in cancer cells promotes metastasis in colon epithelial cells (Ou et al., 2023).

## Enterotoxigenic Bacteroides fragilis

*Bacteroides fragilis* (*B. fragilis*) is a gram-negative anaerobic bacterium that has been identified as a human colonic symbiont (Jo et al., 2023). *B. fragilis* constitutes 1% of the total intestinal microbiome, and 80% of children and adults are tested positive for this bacterium in the intestines (Dadgar-Zankbar et al., 2023). This bacterium is divided into two types: 1) enterotoxigenic *B. fragilis* (ETBF) and 2) non-enterotoxigenic types of *B. fragilis*. *B. fragilis* toxin (*bft*) is a 20 kDa zinc metalloprotease toxin with three isotypes, including 1) *bft-1*, 2) *bft-2*, and 3) *bft-3* (Cheng et al., 2020). Published studies have shown that *bft-2* is more carcinogenic than *bft-1* (Dadgar-Zankbar et al., 2023). ETBF plays a main role in initiating and exacerbating gastrointestinal diseases such as CRC through multiple molecular processes (Qu et al., 2023).

**Figure 5:**
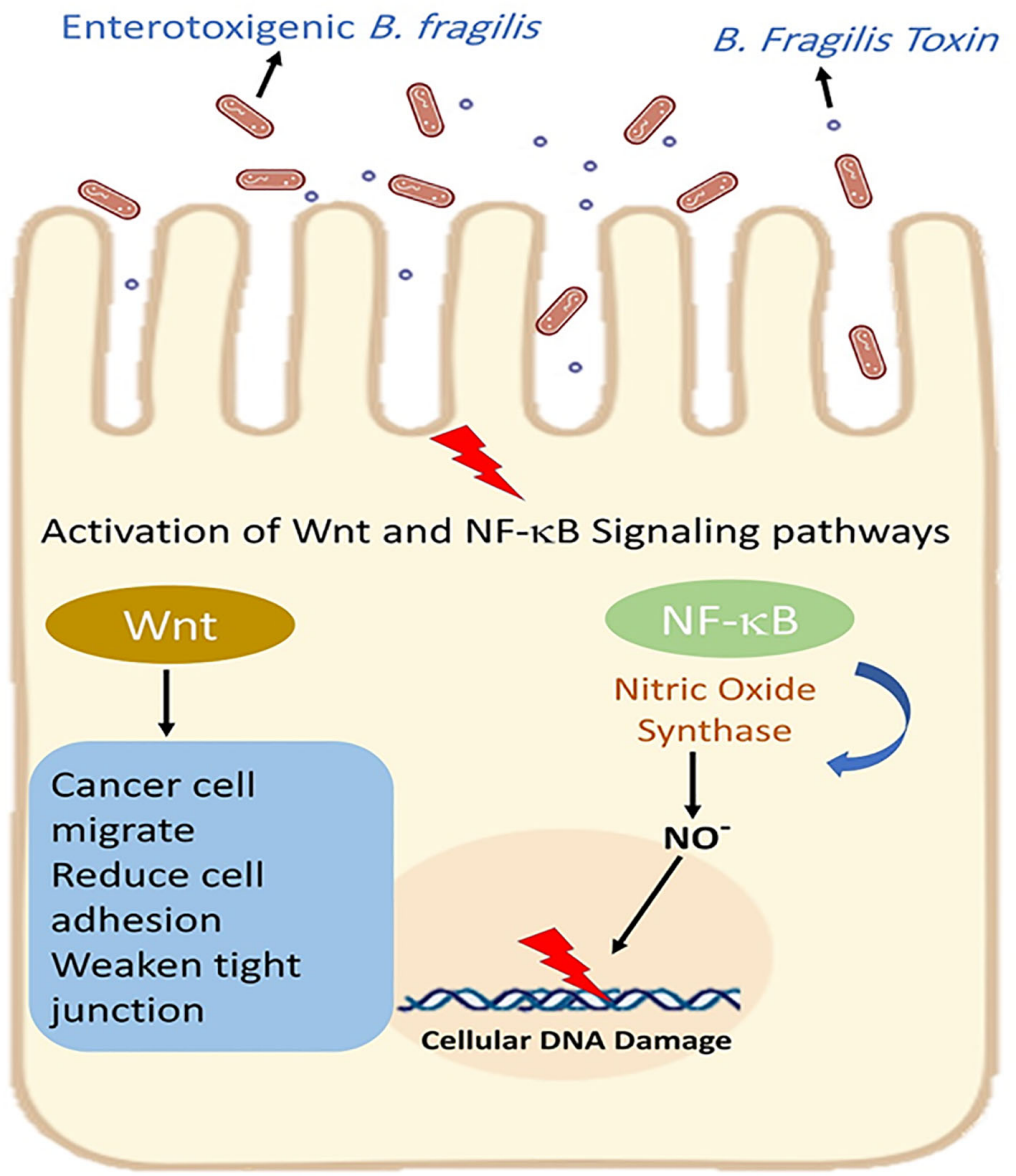
Enterotoxigenic *B. fragilis* increases intestinal secretion and cell signaling via the β-catenin/Wnt pathway. Moreover, BFT activates nuclear factor-κB (NF-κB) and promotes the production of inflammatory cytokines.

**Figure 6:**
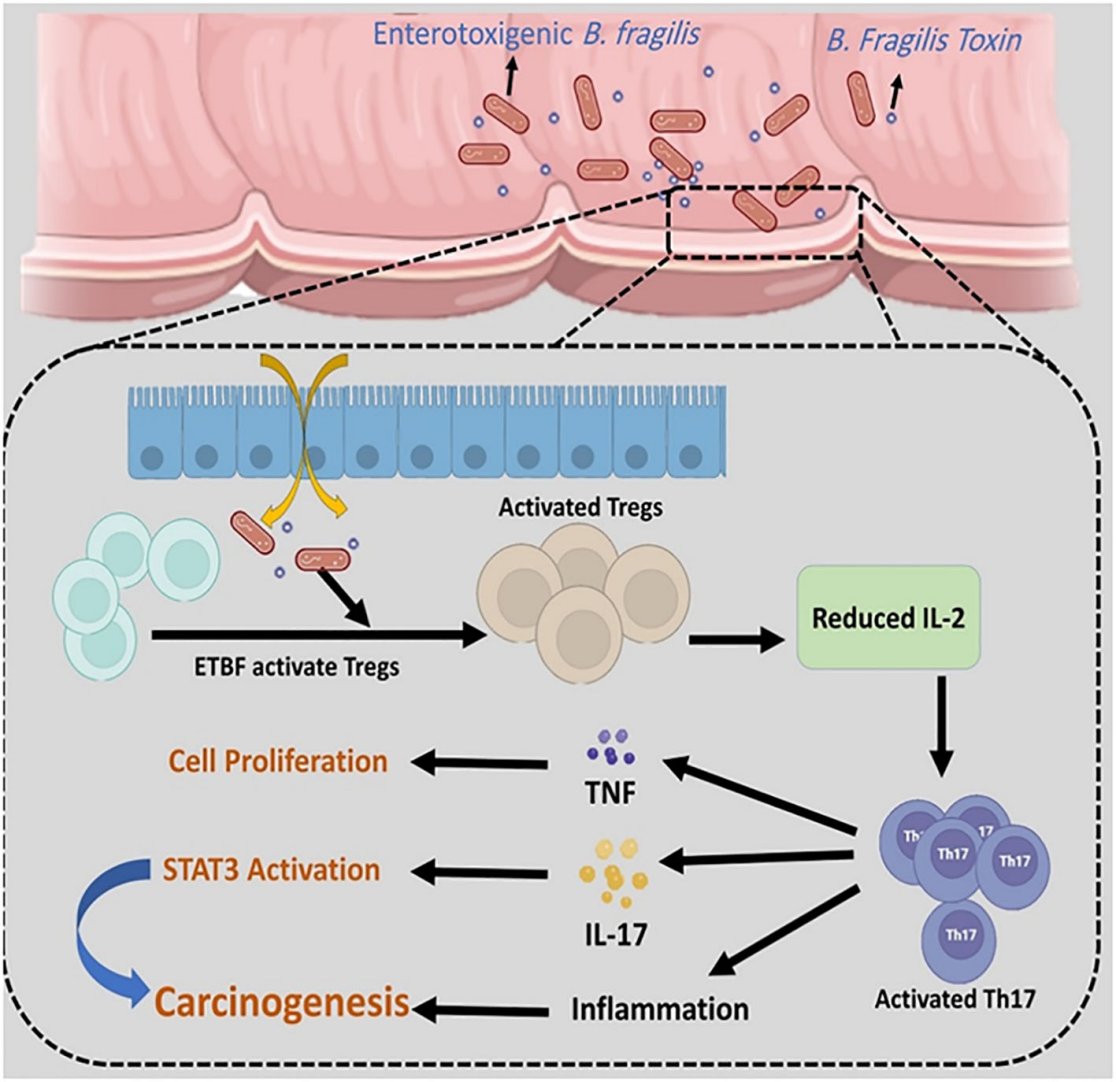
ETBF can activate regulatory T cells (Tregs) and stimulate the high expression of Th17. The overexpression of Th17 leads to an increase in TNF-α, IL-17, and inflammation. STAT3 can act as an oncogene, and constitutively activating it promotes chronic inflammation and tumorigenesis.

E-cadherin is an important regulator of epithelial-mesenchymal transition (EMT) (Tabowei et al., 2022). Both *in vitro* and *in vivo* model studies have revealed that BFT can induce the cleavage of extracellular domain of the E-cadherin junction protein and lead to the disruption of E-cadherin and β-catenin linkage. The disruption of E-cadherin and β-catenin linkage leads to changes in epithelial cell cytoskeletal structure. It increases intestinal secretion and cell signalling via the β-catenin/Wnt pathway. Moreover, the cleavage of E-cadherin activates nuclear factor-κB (NF-κB) and promotes the production of inflammatory cytokines such as CXCL1 (C-X-C Motif Chemokine Ligand 1)(**Figure 5**) (Cheng et al., 2020; Khodaverdi et al., 2021). It is revealed that the disruption of E-cadherin and β-catenin linkage can stimulate the secretion of IL-8 and c-myc expression. C-myc is often persistently expressed in cancer cells. IL-8 can lead to activation of signal transducer and activator of transcription 3 (Stat3; regulators of various cell functions such as acute inflammation). Stat3 can act as an oncogene, constitutively activating it to promote chronic inflammation and tumorigenesis (**Figure 6**). Moreover, studies have stated that Stat3 can skew host immune responses towards tolerance in ETBF infections (Hwang et al., 2013; Purcell et al., 2022). BFT also produces reactive oxygen species (ROS) and causes oxidative DNA damage (**Figure 5**). Besides, ETBF stimulates the high expression of IL-17 and increases the risk of CRC (Khodaverdi et al., 2021). MicroRNAs (miRNAs) are noncoding and single-stranded RNAs with approximately 22 base pairs. Protein translational processes are regulated by miRNAs. Moreover, the molecular and cellular processes in cancer and inflammatory states are greatly influenced by miRNAs (Bartel, 2018; Slack and Chinnaiyan, 2019).

Another carcinogenic process related to ETBF is the downregulation of miR-149-3p. It is revealed that ETBF downregulates the miR-149-3p and promotes intestinal inflammation and malignancy in CRC cells (Cao et al., 2021). Moreover, animal model studies have shown that ETBF can accelerate colitis and carcinogenesis related to dextran sulfate sodium (DSS) and azoxymethane (AOM). However, the molecular mechanism of this process remains unclear (Tabowei et al., 2022).

## Veillonella parvula

*Veillonella parvula* (*V. parvula*) is a strictly gram-negative anaerobic coccus-shaped bacterium belonging to the genus *Veillonella*. This bacterium is a member of normal microbiota in human intestinal, oral, and respiratory tract (Qian et al., 2023). *V. parvula* uses nitrate respiration to colonize the intestine during the inflammatory response. Therefore, *V. parvula* could play a significant role in different digestive diseases such as CRC (Rojas-Tapias et al., 2022). This bacterium increases B lymphocyte stimulator (BLyS) expression level and B-cell infiltration in CRC patients (Qian et al., 2023). Studies have indicated that an increase in BLyS expression level leads to an increase in the level of B cells in tumor tissues (Du et al., 2022; Qian et al., 2023). It is presumed that *V. parvula* can exert an immunomodulatory effect in CRC cells. CRC patients with high fecal *V. parvula* abundance are more likely to develop an advanced tumor stage, higher rates of lymph node metastasis, and a lower prognosis (Qian et al., 2023).

## Prevotella

Colorectal adenomas (CRAs) are precancerous lesions present in 30–40% and <20% of individuals aged ≥ 70 years and ≤ 50 years, respectively[50]. *Prevotella intermedia* (*P. intermedia*) is an anaerobic bacterium that can transform CRAs into colorectal cancer (CRC). Previously published studies have revealed that this bacterium can increase cell migration and invasion in CRC cells[7, 50, 51]. The gut microbial metabolite formate plays a key role in activating aryl hydrocarbon receptor (AhR) signalling [52]. AhR is a ligand-activated transcription factor and a receptor for multiple physiological ligands[53]. Interestingly, *P. intermedia* utilizes several enzymes to produce formate. Furthermore, this bacterium metabolizes glucose for formate formation. The formate produced by *P. intermedia* induces colon cancer cell invasion and leads to metastatic dissemination in CRC[50, 54]. Autocrine motility factor (AMF), also known as glucose-6-phosphate isomerase (GPI), secreted by tumor cells, drives epithelial-mesenchymal transition, and stimulates invasion and metastasis in various cancers, including CRC[55]. It is supposed that *P. intermedia* secretes the AMF molecule and ultimately promotes CRC cell motility and metastasis [56, 57].

## Clostridioides difficile

*Clostridioides difficile* (*C. difficile*) is an anaerobic gram-positive spore-forming bacterium. 5-15% of the adult population is tested positive for this bacterium as a normal gut microbiota (Tirelli et al., 2023). Most patients infected with this bacterium are asymptomatic or have limited to mild diarrhea. However, *C. difficile* can cause different forms of infectious diseases, ranging from infectious diarrhea (antibiotic-associated diarrhea), pseudomembranous colitis, and fulminant colitis (toxic megacolon). Pseudomembranous colitis and fulminant colitis are seen in 25% and 1-3% of patients, respectively (Magat et al., 2020; Tirelli et al., 2023). Several studies have reported that infection by toxin-producing *C. difficile* has a potential role in disruption of gut microbiome and leads to tissue damage and malignant transformation of cells in the colon (Jahani-Sherafat et al., 2019; Kaźmierczak-Siedlecka et al., 2020). This bacterium can produce three toxins, including TcdA (*C. difficile* Toxin A), TcdB (*C. difficile* Toxin A), and cdtA/B (*C. difficile* binary toxin A and B). In comparison to TcdA, TcdB is an important toxin and has more toxic effects on colon cells in *C. difficile* infection (CDI) (Dicks et al., 2019).

It is proven that TcdB toxin induces the inflammatory response, deregulates Rho-GTPases, and increases the expression levels of proto-oncogenes in colon (Jank et al., 2007). Briefly, TcdB binds to its specific receptors, and the toxin-receptor complex enters the host cells by endocytosis. At the next step, TcdB passes from the acidic endosomal membrane and is translocated into the cytosol. Finally, the active part of the TcdB toxin (glucosyltransferase) is released into the cytosol. Glucosyltransferase transfers glucose molecules into Rho proteases and prevents the normal binding of Guanosine-5’-triphosphate (GTP) to Guanosine diphosphate (GDP) bound form of Rho protein (Fettucciari et al., 2023). Therefore, the TcdB toxin can inactivate the Rho protein (Jena et al., 2013; Dicks et al., 2019). Inactivation of the Rho protein stimulates pro-inflammatory interleukin production, tumor necrosis factor (TNF) activation, increases vascular permeability, and finally can lead to the growth and proliferation of tumor cells in CRC cells (Dicks et al., 2019). Phospholipase C-γl (PLC-γl) is an important signalling molecule, and overexpression of PLC-γl is related to cellular transformation (Nam et al., 2012). It is shown that the expression of PLC-γl is upregulated in CRC cells and breast carcinoma. TcdA can induce apoptosis in PLC-γl-transformed cells. Moreover, TcdA can upregulate the expression levels of BIM (a novel member of the BCL-2 family that promotes apoptosis) and activation of caspase-3 in PLC-γl-transformed cells. The high upregulation of PLC-γl by TcdA suggests that this toxin may have good potential as an anticancer agent against different malignancies such as CRC (Warny et al., 2000; Nam et al., 2012).

## Clostridium perfringens

*Clostridium perfringens* (*C. perfringens*) is a gram-positive, anaerobic spore-forming bacterium belonging to the Clostridiaceae family (Huang and Wang, 2020). This bacterium can be isolated from sewage, soil, human genital (reproductive) and urinary systems, and the intestinal tract (Kohya et al., 2022). *C. perfringens* is a causative agent of food poisoning after trauma and gas gangrene. This bacterium could lead to death in a short time in patients with cancer or immunocompromised individuals (Huang and Wang, 2020). *C. perfringens* has the highest frequency in patients with CRC, pancreatic cancer and gastric cancer, respectively (Kohya et al., 2022). First-line treatment for peptic gastrointestinal disorders involves proton pump inhibitors (PPIs). PPIs have been proven to inhibit colon cancer cell growth and carcinogenesis (Zeng et al., 2016; Sasaki et al., 2020). By suppressing the overexpressed protein kinase in T-LAK cells in various cancers, PPIs can inhibit the development and progression of cancer. Furthermore, membrane-bound ATP-binding cassette transporters are suppressed by PPIs, which can reduce drug resistance in cancer (Sasaki et al., 2020). It is revealed that long-term PPI use initiates and exacerbates the *C. perfringens* infection in intestinal tract. *C. perfringens* enterotoxin could impair Claudin-4 (CLDN4), which forms tight junctions in colorectal cells and enhances cancer malignancy (Sasaki et al., 2020). Moreover, this bacterium activates the Yes-associated protein (YAP) and promotes cancer progression (Fujiwara-Tani et al., 2018).

## Clostridium septicum

*Clostridium septicum* (*C. septicum*) is an anaerobic spore-forming gram-positive rod-shaped bacterium belonging to the *Clostridium* family (Lintin et al., 2014). This bacterium is a highly virulent pathogen that produces different toxins and has been linked to hematological malignancy, colorectal malignancy, cyclical neutropenia, diabetes mellitus, and immunosuppression. (Nanjappa et al., 2015) Overall, 50 to 85% of *C. septicum* infections are associated with underlying malignancy (Kennedy et al., 2005). Anaerobic and spore-forming abilities allow *C. septicum* to flourish in the gut. It enters the bloodstream directly after passing through the mucosal layer (Lintin et al., 2014). Nevertheless, the pathophysiology behind how this bacterium promotes colonic malignancy is still unclear. A previously published study pointed out that *C. septicum* and UshA genotoxin in *Escherichia coli* may follow a similar pathogenesis. It is presumed that these bacteria, via DNA damage and defects in cellular DNA repair processes, can lead to carcinogenesis in the colon (Abraham and Padam, 2023). Spore germination, active *C. septicum* infection, and toxin production are done in hypoxic and acidic conditions. Therefore, according to the presence of this condition (anaerobic glycolysis) in tumor tissue, the tumor environment is suitable for the formation of active infection (Jessamy et al., 2016; Justesen et al., 2022). Furthermore, blood supply is relatively low in tumor tissue and this condition could prepare the environment for *C. septicum* proliferation (Justesen et al., 2022). *C. septicum* has several toxins and enzymes such as hyaluronidase, fibrinolysin, deoxyribonuclease, and hemolysins, which enable bacteria to metastasize and invade tissues in the colon (Bickerton et al., 2022).

## Actinomyces

Actinomycetes are a heterogeneous group of gram-positive bacteria commonly found in the human digestive and genital tract, and oral cavity (Fukunaga et al., 2024). The bacteria belonging to the Actinomycetes can cause severe infections in the peritoneal cavity, abdominal wall, face, neck and chest (Acquaro et al., 2010; Valour et al., 2014). *Actinomyces israelii* (*A. israelii*) is a rod-shaped anaerobic gram-positive bacterium and is considered as the most common pathogen among Actinomycetes (Fukunaga et al., 2024). Actinomycosis is a chronic suppurative and granulomatous disease that is caused by *A. israelii* (Valour et al., 2014). A few studies have presumed that *A. israelii* could have a role in initiation or exacerbation of CRC (Fukunaga et al., 2024). There are three conceivable reasons for inflammation and perforation of gastrointestinal tract caused by this bacterium; 1) this bacterium can disrupt the intestinal wall and enter the abdominal cavity; 2) during the surgery for local recurrence, it’s possible that *A. israelii* enters the abdominal cavity; 3) injuries to the oral cavity or gastrointestinal tract could have allowed organisms to enter the bloodstream (Yang and Im, 2018; Fukunaga et al., 2024).

*Actinomyces odontolyticus* (*A. odontolyticus*) is another anaerobic bacterium in human oral cavity and gastrointestinal tract (Könönen and Wade, 2015). Previously published studies have reported that this bacterium is frequently isolated from stool samples of patients with early-stage CRC (Yachida et al., 2019; Breau, 2024). It is revealed that *A. odontolyticus* plays a main role in colorectal dysplasia. This bacterium secretes membrane vesicles (MVs) and induces mitochondrial dysfunction and excessive reactive oxygen species (ROS) production. ROS production can lead to DNA damage and cellular transformation in colonic epithelium (Miyakawa et al., 2024). Furthermore, MVs enriched for lipoteichoic acid (LTA) specifically binds with the TLR2 receptor and activates the NF-kB signaling pathway (Breau, 2024).

## Peptostreptococcus

*Peptostreptococcus anaerobius* (*P. anaerobius*) is an anaerobic gram-positive coccus and is considered as a member of the human oral and gut normal microbiota (Gu et al., 2023). This bacterium can cause several infectious diseases, including endocarditis, periodontal disease, genitourinary and gastrointestinal tract infections (Tsoi et al., 2017). Results of studies have shown that this bacterium was significantly present in stool and mucosal samples of patients with CRC (Yu et al., 2017; Brennan and Garrett, 2019; Cheng et al., 2020). The interaction of *P. anaerobius* with TLR-2 and TLR-4 elevates ROS production in colon cells and leads to CRC tumorigenesis (Tsoi et al., 2017). The increases in the level of intracellular ROS enhance the biosynthesis of cholesterol (Tsoi et al., 2017). Briefly, *P. anaerobius* facilitates the activity of sterol regulatory element-binding protein 2 (SREBP-2). SREBP2 is a key regulator of cholesterol biosynthesis, and this molecule controls the expression levels of genes involved in the synthesis of cholesterol. Therefore, *P. anaerobius* promotes ROS production and cholesterol biosynthesis and enhances cell proliferation and tumorigenesis in CRC (Tsoi et al., 2017; Huang et al., 2024).

**Figure 7:**
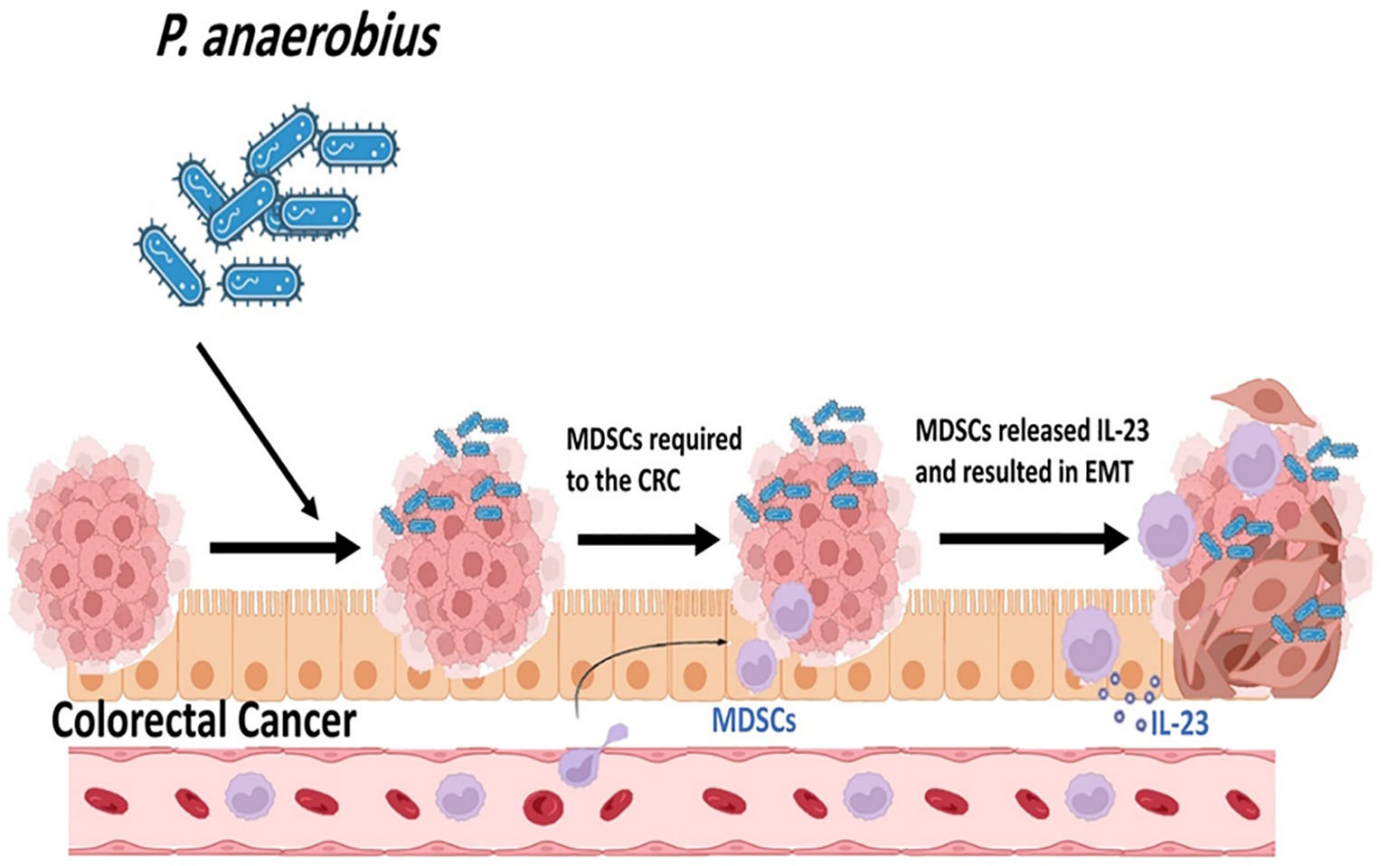
*P. anaerobius* colonization increases Myeloid-derived suppressor cells' (MDSCs) abundances in tumor microenvironment.

Myeloid-derived suppressor cells (MDSCs) are known as the main immune suppressant cells in tumor microenvironment and are linked to cancer progression (Liu et al., 2024). It is presumed that the intratumoral bacteria play a main role in accumulation of MDSCs in CRC cells (Gu et al., 2023). Results of a study performed on the mouse models revealed that *P. anaerobius* colonization increases MDSCs abundances in tumor microenvironment (**Figure 7**) (Liu et al., 2024). The presence of *P. anaerobius* in tumor microenvironment stimulates the secretion of chemokine (C-X-C motif) ligand 1 (CXCL1) (Long et al., 2019; Liu et al., 2024). CXCL1 is a main chemokine in the recruitment and activation of neutrophils for microbial killing. This chemokine plays a main role in host immune response. CXCL1 participates in the chemotaxis of CXCR2 (C-X-C Motif Chemokine Receptor 2) + MDSCs. The accumulation of MDSCs in tumor microenvironment can antagonistically affect antitumor CD8+ and CD4+ T cells (Long et al., 2019; Gu et al., 2023). Moreover, it is revealed that the recruitment of MDSCs into tumor microenvironment increases the chemoresistance of colorectal cancer to oxaliplatin and mediates anti-PD1 therapy resistance (Liu et al., 2024).

The putative cell wall binding repeat 2 (PCWBR2) is a surface protein of *P. anaerobius*. The direct interaction of PCWBR2 with epithelial cell receptor integrin α2/β1 leads to the activation of B cells (NF-κB) cascade and ultimately promotes cell proliferation and pro-inflammatory immune responses in CRC cells (Long et al., 2019).

**Figure 8:**
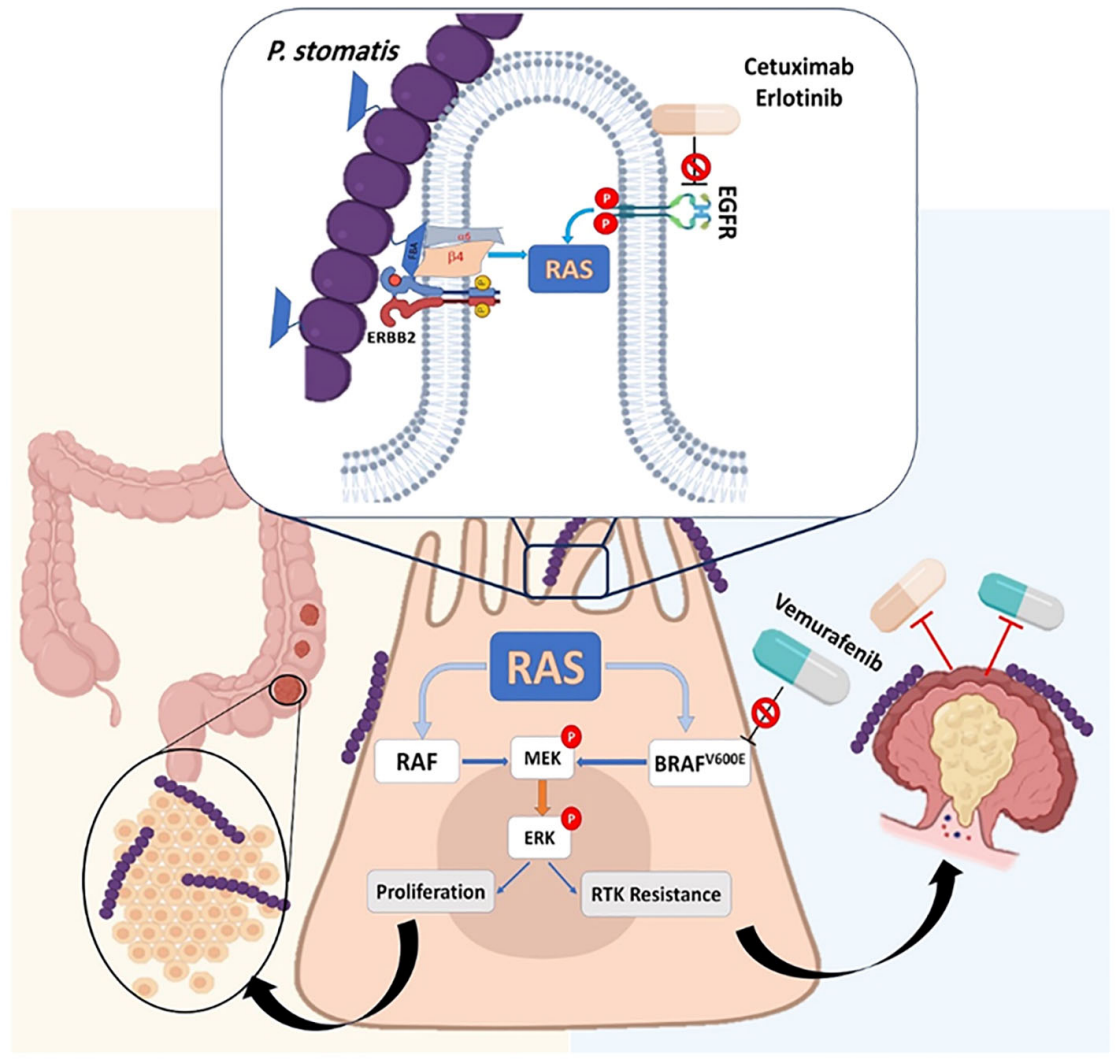
*P. stomatis* can activate the erb-b2 receptor tyrosine kinase 2 (ERBB2)-mitogen-activated protein kinase (MAPK) and promote colonic tumorigenesis. This bacterium applies its surface protein fructose-1,6-bisphosphate aldolase (FBA) to attachment on the CRC cells. The attachment of *P. stomatis* to CRC cells leads to the activation of ERBB2 and downstream Ras/Raf/MAPK (MEK)- extracellular signal-regulated kinase (ERK) cascades. *P. stomatis* can inactivate and inhibit the BRAF inhibitor (a member of RAF serine/threonine kinases) (vemurafenib) and receptor tyrosine kinase (RTK) inhibitor (cetuximab, erlotinib), leading to non-responsiveness to these drugs in CRC.

*Peptostreptococcus stomatis* (*P. stomatis*) is another species of *Peptostreptococcus* spp. that is frequently isolated from patients with CRC (Osman et al., 2021). Results of a previously published animal model study indicated that *P. stomatis* can activate the erb-b2 receptor tyrosine kinase 2 (ERBB2)-mitogen-activated protein kinase (MAPK) and promote colonic tumorigenesis (Huang et al., 2024). This bacterium applies its surface protein fructose-1,6-bisphosphate aldolase (FBA) to become attached to CRC cells. The attachment of *P. `stomatis* to CRC cells leads to the activation of ERBB2 and downstream of Ras/Raf/MAPK (MEK)- extracellular signal-regulated kinase (ERK) cascades (**Figure 8**) (Shen et al., 2021). Moreover, it is revealed that *P. stomatis* can inactivate and inhibit the BRAF inhibitor (a member of RAF serine/threonine kinases) (vemurafenib) and receptor tyrosine kinase (RTK) inhibitor (cetuximab, erlotinib), leading to the non-responsiveness to these drugs in CRC. This bacterium can suppress apoptosis and impair the gut barrier function in mouse models of CRC (Osman et al., 2021; Huang et al., 2024).

## Conclusion

In general, the exact role of anaerobic gut bacteria in initiation, exacerbation and development of human CRC is not completely identified. In the present study, we searched various databases and gathered the results of previously published studies and clinical and epidemiological signs and the possible mechanisms of some anaerobic gut bacteria in CRC were shown. Different studies have revealed that many anaerobic gut bacteria including *Fusobacterium nucleatum*, *C. difficile*, Enterotoxigenic *B. fragilis*, *P. anaerobius*, *P. stomatis*, etc. could contribute to initiation, development, and exacerbation of CRC in animal models and human. However, more animal models and clinical trial studies are required for a precise conclusion regarding the role of anaerobic gut bacteria in CRC development.
